# Harnessing Synergistic Biostimulatory Processes: A Plausible Approach for Enhanced Crop Growth and Resilience in Organic Farming

**DOI:** 10.3390/biology11010041

**Published:** 2021-12-28

**Authors:** Md. Nasir Hossain Sani, Jean W. H. Yong

**Affiliations:** 1 School of Natural Sciences, Bangor University, Bangor LL57 2DG, UK; 2 Department of Biosystems and Technology, Swedish University of Agricultural Sciences, 234 56 Alnarp, Sweden

**Keywords:** plant biostimulants, synergistic effect, nutrient use efficiency, abiotic stress, crop resilience, organic farming, phytohormones, microbes

## Abstract

**Simple Summary:**

Demand for organically grown crops has risen globally due to its healthier and safer food products. From a sustainability perspective, organic farming offers an eco-friendly cultivation system that minimizes agrochemicals and producing food with little or no environmental footprint. However, organic agriculture’s biggest drawback is the generally lower and variable yield in contrast to conventional farming. Compatible with organic farming, the selective use of biostimulants can close the apparent yield gap between organic and conventional cultivation systems. A biostimulant is defined as natural microorganisms (bacteria, fungi) or biologically active substances that are able to improve plant growth and yield through several processes. Biostimulants are derived from a range of natural resources including organic materials (composts, seaweeds), manures (earthworms, fish, insects) and extracts derived from microbes, plant, insect or animal origin. The current trend is indicative that a mixture of biostimulants is generally delivering better growth, yield and quality rather than applying biostimulant individually. When used correctly, biostimulants are known to help plants cope with stressful situations like drought, salinity, extreme temperatures and even certain diseases. More research is needed to understand the different biostimulants, key components, and also to adjust the formulations to improve their reliability in the field.

**Abstract:**

Demand for organically grown food crops is rising substantially annually owing to their contributions to human health. However, organic farm production is still generally lower compared to conventional farming. Nutrient availability, content consistency, uptake, assimilation, and crop responses to various stresses were reported as critical yield-limiting factors in many organic farming systems. In recent years, plant biostimulants (BSs) have gained much interest from researchers and growers, and with the objective of integrating these products to enhance nutrient use efficiency (NUE), crop performance, and delivering better stress resilience in organic-related farming. This review gave an overview of direct and indirect mechanisms of microbial and non-microbial BSs in enhancing plant nutrient uptake, physiological status, productivity, resilience to various stressors, and soil-microbe-plant interactions. BSs offer a promising, innovative and sustainable strategy to supplement and replace agrochemicals in the near future. With greater mechanistic clarity, designing purposeful combinations of microbial and non-microbial BSs that would interact synergistically and deliver desired outcomes in terms of acceptable yield and high-quality products sustainably will be pivotal. Understanding these mechanisms will improve the next generation of novel and well-characterized BSs, combining microbial and non-microbial BSs strategically with specific desired synergistic bio-stimulatory action, to deliver enhanced plant growth, yield, quality, and resilience consistently in organic-related cultivation.

## 1. Introduction

The pressing concern of global food security combined with projections of global population increase and climate change poses a major threat to agriculture in terms of reliability, sustainability, maximizing productivity while minimizing the agro-ecosystems’ environmental impact. The current global population is expected to rise from approximately 7.5 billion to over 9.7 billion by 2050 [1]. Consequently, global requirement for food crops was projected to rise by at least 60% by 2050 [2]. The green revolution has increased agricultural productivity by intensifying food production through the extensive use of chemical fertilizers, agrochemicals, and modified crop varieties. However, in many cases, commercialization of these practices has a substantial impact on soil health, inevitably leading to hazardous environmental consequences. The prolonged and extensive usage of agrochemicals and fertilizer has inevitably led to soil degradation, soil acidification, depletion of essential soil nutrients, groundwater contamination, eutrophication of waterways, and greenhouse gas emissions [3,4]. Recent studies reported that approximately 1% of chemical pesticides actually reach their target sites, and remaining amount resided in the environment [5,6]. Besides, loss of beneficial microbial populations from soil is one of the serious long-term impacts of agrochemicals in the soil ecosystem [7,8]. In this context, organic farming, which restricts the use of agrochemicals, has drawn tremendous consumer attention and scientific interest.

Over the last decades, consumers’ interest in organically grown crops has risen worldwide due to its healthier and safer products [9,10,11]. Furthermore, organic farming offers an eco-friendly production system that minimizes off-farm inputs and minimal damage to the ecosystems [9,12]. However, organic agriculture’s biggest drawback is the generally lower yield in contrast to intensive farming [9,13]. Several meta-analyses reported 8–25% lower yield in organic farming than intensive farming [13,14,15]. Therefore, more land is required to produce the same yield return, which in many instances contribute to greater land-use requirements (e.g., deforestation) and, consequently, outweigh the environmental benefits of organic farming practices [9,16]. The major yield-limiting factor in organic farming is associated with nutrient bioavailability, uptake, and assimilation owing to slow and/or inconsistent release of nutrients from various organic inputs [11,17]. Furthermore, biotic pressures (both fungal and bacterial diseases) were reported to cause substantial yield reduction in some organic production systems [18,19]. Apart from this, the rapidly changing climate poses environmental constraints, including drought, temperature, and salinity stresses [20,21]. Genetically modified (GM) crops remain a feasible option to overcome such limitations. However, research and regulations required to produce resistant varieties through traditional breeding approaches would take decades to reach the market place with formal approvals [22]. Therefore, to address these contemporary challenges in an environment-friendly, practical, and sustainable way, organic farming practices seek innovative solutions focusing on nutrient use efficiency (NUE), consistency in nutrient availability, abiotic stress tolerance, crop yield, and quality [9,23,24].

A plausible, innovative and organic farming compatible technology would be the use of plant biostimulants (BSs) that have recently gained much interest globally [21,25,26,27,28,29]. According to du Jardin [30], BSs are naturally occurring compounds that stimulate plant physiological and molecular processes and thereby modulating crop yield and quality. However, there is no legal framework globally for defining BSs from a regulatory perspective [31,32]. Nonetheless, the global BSs market continues to grow rapidly, surpassing €2.7 billion by 2022, propelled by many governments’ increasing focus on improving sustainability while reducing the environmental footprint of food production [33]. Moreover, the estimated demand for organic food products is over US$300 billion by 2022, with simultaneous increase in organic farmland of 75 million hectares by 2020 [33].

In recent years, advancement in biochemical, genomic, and transcriptomic tools significantly contributed to unveiling the mode of actions of BSs [27,34,35,36,37]. This advancement has opened the doors for many BSs related industries to look for more effective and reliable formulations by blending microbial BSs with non-microbial BSs. However, many of these approaches were implemented without having proper scientific evaluation. Recent literature reported that the purposeful combinations of microbial and non-microbial BSs would interact synergistically and enhance growth and yield over a single application [24,32,33]. Designing target-specific BSs formulations would be pivotal for increasing NUE, consistency in nutrient availability, crop growth, and resilience in supporting a renaissance in organic farming. However, little is known about the interactive effects between microbial and non-microbial BSs, their ecological effects on rhizosphere microbes, rhizosphere, and plant metabolic dynamics. This review examined bio-stimulatory actions/mechanisms, interactive effects of microbial and non-microbial BSs affecting growth, and resilience to environmental stresses. It also discussed the scientific progress made in microbial and non-microbial BSs formulations and their performance in various fields and greenhouse experiments. The review also identified issues hindering improvements in crop yield and resilience as the world moves towards meeting the challenges of sustainable farming.

## 2. Microbial and Non-Microbial Biostimulants: Action/Mechanisms and Biostimulatory Effects on Plants

**Figure 1:**
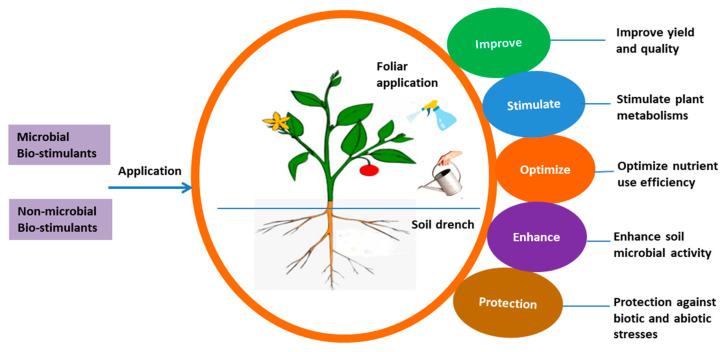
Bio-stimulatory effect of microbial and non-microbial biostimulants on different aspects of plant growth and productivity; Adapted from [40].

**Figure 2:**
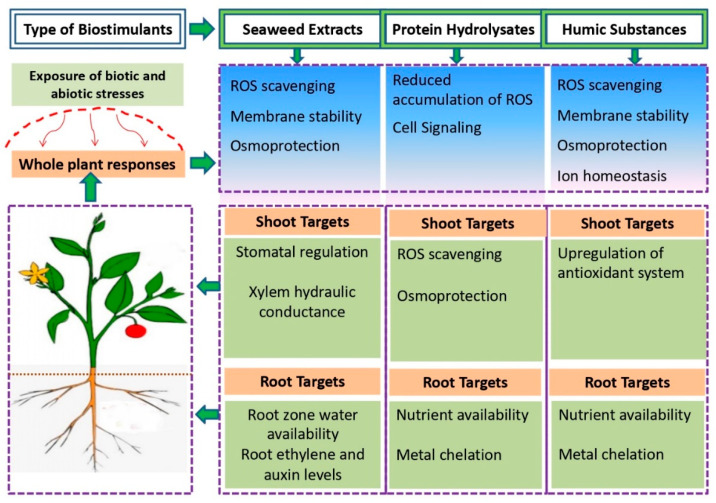
Key bio-stimulatory mechanisms targeted by non-microbial biostimulants upon interaction with plants and their growing environment; adapted from [86,87].

**Figure 3:**
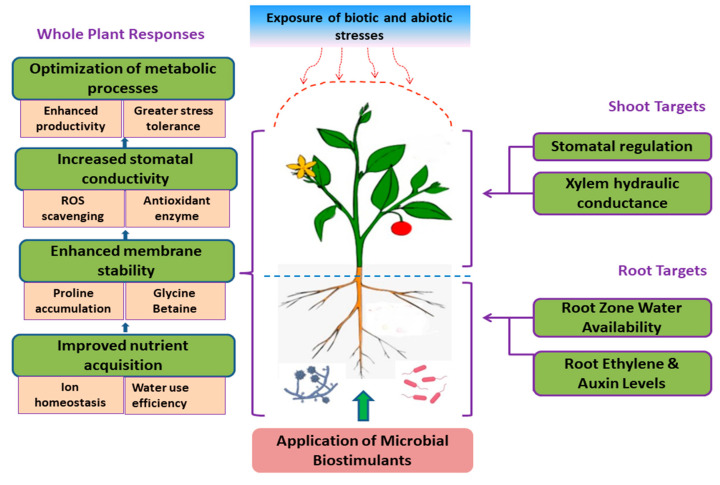
Key plausible bio-stimulatory mechanisms targeted by microbial biostimulants upon interaction with plants and their growing environment.

Bio-based products such as organic BSs render a sustainable, effective technology for enhancing NUE and ensuring a stable yield of agricultural and horticultural crops under optimal and sub-optimal conditions [24,26,32,34,35,36,37,38]. Non-microbial organic BSs include humic substances (HSs), protein hydrolysates (PHs), and seaweed extracts (SWEs). Besides, bacterial-based BSs, including diverse species of PGPRs (*Azotobacter*, *Azospirillum*, and *Rhizobium* spp.) and fungal-based BSs (*Trichoderma* spp., *mycorrhizal fungi*) have been promoted as promising microbial BSs for enhanced crop productivity and stress tolerance in numerous crops [29,38,39,40]. Based on scientific literature, we collated the different effects of BS application on various agronomic, physiological, biochemical, and molecular aspects of plant growth, productivity, quality, and resilience (Figure 1). Apart from the bio-stimulatory effects of BSs on crops and other species, it was important to shed more light on the key mechanisms of non-microbial BSs (Figure 2) and microbial BSs (Figure 3) involved in regulating physiological and other metabolic processes, leading to better NUE, growth and resilience. 

### 2.1. Non-Microbial Plant Biostimulants

#### 2.1.1. Humic Substances 

Humic substances (HSs) are natural soil organic substances derived from plant, animal, microbial decomposition, and the metabolic activity of soil microbes. These heterogeneous compounds exhibit complex dynamics with soil microbes that are influenced by plant roots and their exudates. The interactivity of HSs, plant roots, and rhizosphere microbes combined generally to promote plant growth and yield. In fact, HSs are well recognized for their long-term contribution to soil fertility through enhanced physical, chemical, and biological attributes. The most widely reported bio-stimulatory action of HSs is the enhanced macro- and micro-nutrient uptake through increased cation exchange capacity of soil, known commonly as the HS-facilitated root nutrition. The stimulation of plasma membrane H+ ATPases transformed free energy produced by ATP hydrolysis into a transmembrane electrochemical potential that is used for importing nitrate and other nutrients [41,42]. HSs are known to interact with calcium-phosphate precipitation and thereby increasing phosphorus solubility for plant uptake [42,43]. Additionally, HSs were reported to regulate reactive oxygen species (ROS) concentration and superoxide dismutase (SOD) genes in cytosol, promoting cell growth and differentiation [44]. The biostimulatory actions of HSs were found to be effective in increasing root uptake of sulphate as well as gene expression of primary sulphate transporters in roots [45]. Interestingly, HSs were involved in triggering the signaling pathways mediated by auxin and nitric oxide, along with up-regulation of numerous auxin-regulated genes in roots [42,46]. Apart from this, HSs could enhance key enzyme activity due to their higher molecular masses that could modulate stress responses [47,48]. HSs were able to reduce hydrogen peroxide, and lipid peroxidation, thereby increasing proline content and favoring a stress-responsive microbial community in rhizosphere, especially against salinity and drought [49,50]. 

#### 2.1.2. Protein Hydrolysates 

Protein hydrolysates (PHs) are a complex group of compounds derived from the chemical and enzymatic protein hydrolysis of agro-industrial and household byproducts of plant and animal origins [25,34,51]. Animal sourced PHs include leather byproducts, fish byproducts, and chicken feathers, whereas plant origin PHs include vegetable byproducts, legume seeds, alfalfa hay, etc. [52]. Recently, PHs obtained from fish-waste and other aquaculture byproducts have become popular in various communities and industries due to the eco-friendly approach to waste disposal and contributions to circular bioeconomy [53]. According to Colla and co-workers [54], PHs represented a vital category of organic non-microbial BSs with a mixture of active compounds such as amino acids, oligopeptides, polypeptides, etc. These compounds could act directly or indirectly as signaling molecules triggering numerous physiological and molecular processes in plants; thus enhancing growth, and mitigating the adverse effects of abiotic stressors on crops [24,35,36,54,55,56]. PHs stimulate carbon and nitrogen metabolisms, activating key enzymes involved in N uptake and assimilation [30,55]. Interestingly, some phytohormones were found in certain type of PHs; while other PHs showed hormone-like (mainly auxin related) activities in bioassays [24,54,55,56]. Furthermore, PHs contained bioactive peptides, that were reported to stimulate hormonal activities [24,55,56]. Several greenhouse and open-field experiments demonstrated that commercial PHs were able to elicit hormone-like actions (auxin and gibberellins) and fostered favourable root and shoot development leading to better crop productivity [35]. In addition, PHs were reported to modulate root architecture especially root hair development and improving nutrient uptake [54]. 

#### 2.1.3. Seaweed Extracts

Seaweed extracts (SWEs), predominantly brown seaweed extracts, are widely used BSs for growth promotion and mitigating abiotic stress such as salinity, drought, and extreme temperatures in many agricultural and horticultural crops. The commercial SWEs from brown seaweed contain a complex mixture of polysaccharides, fatty acids, phytohormones (auxins, cytokinins, gibberellins, abscisic acid, and brassinosteroids), vitamins, mineral nutrients, and a diverse range of organic components [57,58,59]. SWEs also contain various osmolytes such as betaines that play a crucial role in osmotic and temperature stress tolerance in plants [60,61]. Researchers are working actively on discovering the diversity of bioactive compounds in SWEs and elucidating their bio-stimulatory actions/mechanisms in plants [57,58,59,60,61]. Using advanced analytical tools (metabolomics and transcriptomics approach), researchers discovered alga-specific polysaccharides, betaines, polyamines, phenolic compounds, and phytohormones; these compounds could regulate several gene expression and signaling pathways that are responsible for many observed effects on plants [58,59,60,61]. For instance, SWEs were reported to regulate the nitrate transporter gene “NRT1.1.” which has a significant role in N uptake and assimilation [62]. Furthermore, Khan and co-workers [63] reported that *Ascophyllum nodosum* SWEs activated the nodC bacterial gene and triggered bacteria-plant signaling by mimicking the effect of a flavonoid, luteolin. The triggering of flavonoid production by SWEs plays a substantial role in regulating plant development and responses to UV light and other environmental stresses, although the precise mechanism remains unclear [64]. In addition, the constituents of SWEs were able to induce root colonization of beneficial fungi in rhizosphere [65]. A recent molecular study highlighted the possible mechanisms of SWEs in regulating plant growth and development through hormonal homeostasis, biosynthesis of new transporters for nutrient uptake and assimilation, stimulating photosynthesis, and stress tolerance [66]. Moreover, SWEs were able to promote antioxidant stimulation whilst reducing lipid peroxidation under abiotic stress and contributing to the scavenging of reactive oxygen species (ROS) [58,59,61].

#### 2.1.4. Bioconversion Compost-Derived Biostimulants

Apart from the well-defined categories of BSs, bioconversion of organic by-products that exhibit bio-stimulatory activity could also be classified as a new sub-category within existing ones, as well as creating additional avenues for waste and by-product management [9,27,28,67,68,69,70]. Multiple studies have demonstrated that bioconversion composts, such as earthworms (vermicompost) and larvae-based (e.g., mealworms, black soldier fly) bioconversion compost, produced a substantial number of bioactive compounds that have a wide range of beneficial impacts on plant growth, soil attributes, and alleviation of abiotic stresses [28,68,70,71]. The bio-stimulatory activity of vermicompost is due to a combination of plant hormones, cytokinins, auxins, abscisic acid, gibberellins, brassinosteroids, and other beneficial compounds yet-to-be-identified. For example, numerous cytokinin types have been identified and quantified in vermicompost using advanced analytical technique of mass spectrometry [28,72]. These include zeatin (Z) and isopentenyladenine (iP) classes of cytokinins, which serve as positive phytohormonal signals and guide the plants to advance through key cell cycle checkpoints culminating in increased cell proliferation and growth [28,73,74,75]. Earthworms’ faeces (vermicompost) have been shown to contain iP-type cytokinins due to the presence of gut microbiota in the digestive system, which are antecedents of Z-type cytokinins [28,76]. Interestingly, coconut water was reported to contain a significant amount of Z-type cytokinins and other phytohormones, which have the potential to regulate plant growth as BSs [77,78,79]. Research has specifically linked cytokinins and auxins found in coconut water to a variety of beneficial effects, including plant growth biostimulation, through the regulatory role in plant cell cycle and signaling pathways via several critical molecular checkpoints [73,74,80]. In light of these findings, it was suggested that plants could obtain additional cytokinins in addition to those produced endogenously by directly enriching the soil with a phytohormone-based BSs product. Furthermore, other phytohormones, such as gibberellins, auxins, and brassinosteroids, are anticipated to boost growth-related physiological effects when vermicompost is applied to the soil matrix [28,70,71,81]. The presence of humic and fulvic acids in vermicompost boosted nutrient absorption and activated membrane-associated signal transduction cascades that govern plant growth and development [70,82]. The biostimulatory action of betaines derived from seaweed extracts was also well-established in scientific literature [59,60]. Recently, Huang and co-workers [83] discovered the existence of several betaines in vermicompost and larval bioconversion compost. Betaines have been demonstrated in numerous trials to play a critical role in stabilizing enzyme and protein structure, improving the protection of lipids and membranes and enhancing plants’ resilience to various stressors [84,85].

### 2.2. Microbial Plant Biostimulants

#### 2.2.1. Fungal-Based Microbial Biostimulants

During the evolution of terrestrial plants, plants and fungi have co-evolved together; fungi interact with plant roots in numerous ways, ranging from mutualistic to parasitic [88,89]. Several scientific studies demonstrated that the parasitism-mutualism continuum is beneficial for maintaining ecosystem balance and increasing crop growth and productivity [29,90,91,92,93]. According to leading BSs researchers [30,34,51], the biostimulants’ classification includes fungal-based products used on plants for promoting nutrient uptake efficiency, stress tolerance, crop performance, and product quality. The widely known fungal BSs are *Trichoderma* spp. and various mycorrhizal fungi; well known to increase nutrient uptake and plant growth in an environmentally-friendly way [24,56,94,95,96,97]. Commercially, *Trichoderma* spp. have gained much interest as “effective” microbial BSs due to their multifunctional role in mitigating biotic and abiotic stresses on crops [24,94,95]. Several research groups reported that *Trichoderma* species improved root to shoot signaling by stimulating the biosynthesis of several hormones; these included enhancing nutrient solubility, uptake, assimilation, and leading to higher crop productivity [24,56,65,94,95]. Arbuscular mycorrhizal fungi (AMF), another commonly used microbial BSs, form interesting symbiotic associations with more than 90% of plant species and economically important crops [88,89,94,95]. Generally, AMF provide widespread benefits in sustainable agriculture by improving the nutrient exploration in soil matrices, nutrient uptake and maintaining ion homeostasis under normal and stressful conditions. Although beneficial fungi and their products are widely used as BSs to enhance growth, productivity, and resilience to environmental stresses [65,88,94,95,96,97], the complexity in their interactions with other soil microbes do make it difficult to determine their host-specific bio-stimulatory functions, interactions and nutrient dynamics within any agro-ecosystems [16,29,56,94,95,96,97].

#### 2.2.2. Bacterial-Based Microbial Biostimulants

Bacterial-based BSs are formulations of microbial-derived compounds and diverse groups of plant growth-promoting Rhizobacteria (PGPR) and other beneficial bacteria (e.g., *Actinomycetes*) that promote root development, growth, and stress tolerance [2,29,39,98]. The bacterial taxa commonly used as microbial BSs include *Acetobacter*, *Agrobacterium*, *Azospirillum*, *Azotobacter*, *Bacillus*, *Burkholderia*, *Enterobacter*, *Frankia*, *Pseudomonas*, *Rhizobia*, *Serratia*, and *Streptomyces* [39,75,98,99,100,101,102,103,104]. Applications of microbial BSs were reported to alter several metabolic processes, influence ion homeostasis, enhance water holding capacity, and strengthen antioxidant defense mechanisms, thus delivering better plant growth and resilience [29,51,98,99]. The bio-stimulatory effect of bacterial-based microbial BSs under both normal and stress conditions could be attributed to various direct and indirect actions/mechanisms: (i) enhancing nutrient availability in soil, plant uptake and assimilation; (ii) modulation of root system architecture; (iii) improving water relations and photosynthetic efficiency; (iv) strengthening the antioxidant defense system; (v) production and regulation of phytohormones (auxins, ABA, cytokinins, ethylene, and gibberellins, etc.); (vi) promoting nutrient transporters (NRT1.1, NRT2, NAR2.2, AMT, Pht1, and PT2-1); and (vii) modulation of soil microbiome through enzymes and organic compounds [24,39,75,98,99,100,101,102,103,104,105].

## 3. Implications of Biostimulants for Enhancing Plant Nutrition in Organic Farming

### 3.1. Soil Nutrient Availability 

Organically grown crops are often subjected to nutrient shortages attributed to low soil nutrient levels or to poor nutrient solubility within rhizosphere. Increasing nutrient availability and improving utilization efficiency, especially N and P, are critical for growers operating in this “low-input” cultivation system. The use of bioactive natural substances and inoculants, commonly defined as BSs, would be a valuable method to increase NUE in organic agriculture [9,11,24,39,75,94,95,96]. Operating within any organic production system (“low input” system), the strategic usage of selected BSs will improve nutrient availability by enhancing cation exchange capacity (CEC), thereby increasing the solubility of nutrients in soil for plants’ uptake [42,55,82]. HSs have been reported to enhance soil physico-chemical attributes, consequently increasing essential soil nutrient availability [42,55]. Furthermore, HSs increase CEC and buffer soil pH, which facilitated certain nutrients to become available [42,82]. HSs could also form soluble HSs complexes with trace elements that were identified as a sustainable strategy to prevent micronutrient leaching, thus enhancing their availability for plant uptake [82,106,107]. Several research documented that HSs activated the H+-ATPase plasma-membrane, thereby increasing radical H+ extrusion and reducing root surface pH, which facilitated increased soil nutrient availability for enhanced absorption and translocation [42,82]. PHs also improved soil nutrient availability by forming complexes and chelates between peptides and micronutrients, thus facilitating root-zone nutrient availability [30,52,54,55]. Moreover, PHs provide microbes with amino acids and peptides, thus enhancing substrate availability for microbes, soil respiration and promoting microbial activity, leading ultimately to better nutrient availability [108]. SWEs are another group of organic BSs containing alginates that were reported to be effective soil-conditioners and able to form high molecular mass polymers via metal chelation [109,110]. Furthermore, it was reported that these cross-linked polymeric networks would enhance the soil’s water-holding capacity, promoting root growth and microbial activity and thereby improving nutrient availability in soil [110]. Apart from the non-microbial BSs, microbial BSs such as PGPR were able to promote plant growth through improving nutrient availability, especially for N, P, and Fe [98,99,100]. In addition, through the production of organic acids, PGPR could enhance solubility of phosphates in both organic and conventional systems [98,111]. Fungal-based BSs *Trichoderma* species were able to enhance iron solubility by producing siderophores, thus enhancing plant nutrient uptake [94,95,98,112]. Similarly, AMF could develop extensive hyphal networks to enlarge the surface area for nutrient exploration and uptake and producing organic substances that could solubilize P [95,96,113,114]. Moreover, AMF indirectly increased nutrient availability by improving soil aggregate stability, enhancing cation retention, especially Ca^2+^ and Mg^2+^, and improving the nitrification process [115].

### 3.2. Plant Nutrient Uptake 

Plant nutrient uptake is influenced by a range of factors, including plant species, physiological status, environmental conditions, root growth, and root-associated microorganisms [98,107,116]. In a typical organic farming system, root growth plays a vital role in nutrient acquisition where soil nutrient is usually available at comparatively lower concentrations. In this context, the extensive and vigorous root growth, with optimal root architecture is imperative for ensuring sufficient nutrient uptake to meet crop nutrient demand in this “low-input” farming system. Several studies demonstrated that BSs such as HSs, PHs, and SWEs could foster better root growth and development, thereby facilitating the exploration of more soil matrices for plant nutrient uptake [24,117]. BSs are effective not only for stimulating root growth but can also increase the amount of nutrients absorbed by plants. For instance, studies have demonstrated that HSs not only increased the bioavailability of micronutrients under nutrient-limited conditions but also able to enhance root’s capacity to absorb micronutrients from soil solution [106,107,118]. According to Colla and co-workers [52], PHs stimulated root growth in many crops such as tomato, lettuce, corn, etc. Similarly, for another group of commonly used BSs, the SWEs were effective in stimulating root growth in cuttings [119]. Furthermore, it was also reported that polysaccharide-enriched SWEs promoted stronger root growth-promoting action through several processes: triggering of signaling molecules, changes in endogenous phytohormone metabolism, and up-regulation of selected metabolic genes [120]. Interestingly, some BSs can stimulate specific enzyme activity and promote micronutrient uptake. For instance, PHs enhanced the Fe (III)-chelate reductase activity in both roots and leaves, leading to Fe’s uptake and assimilation under Fe deficient conditions [121].

Several researchers have reported that microbial BSs such as AMF and *Trichoderma* spp. stimulated root growth by producing auxin-like compounds, which promoted root formation [94,95,96,97]. A recent meta-analysis of 52 published PGPR articles reported that PGPR generally increased root biomass by 35% and 43% under well-irrigated and water deficit conditions, respectively [122]. The increased root growth triggered by AMF and PGPR allows plants to explore more soil matrices, thus strengthening plants’ capacity to cope with possible low nutrient and soil water availability situations. Moreover, HSs were able to upregulate several nutrient transporter genes such as the nitrate transporters (BnNRT1.1 and BnNRT2.1) and sulfate transporters (BnSultr1.1 and BnSultr1.2), thereby increasing nitrogen and sulfate uptake by plants [45]. Apart from non-microbial BSs, microbial BSs such as AMF and PGPR were reported to upregulate the nitrate transporter gene (NRT1.1, NRT2, and NAR2.2) expressions and subsequently increase the nitrogen uptake [105].

### 3.3. Plant Nutrient Assimilation

BSs can promote the assimilation of nutrients (for example, nitrate, ammonium, phosphate, and sulfate) directly through inducing gene expression of plant metabolism enzymes; and indirectly by increasing nutrient absorption and transport. Jannin and co-workers [45] conducted a microarray analysis of 31,561 genes. They demonstrated that 300 genes were expressed after three days following HSs’ application, whereas the numbers were reduced to 102 genes after 30 days. Among them, 80% of the genes were related to sulfate metabolism and these were upregulated by HSs. The bio-stimulatory actions of PHs were also reported to stimulate enzymatic activity of carbon metabolism (malate dehydrogenase, citrate synthase, etc.) and as well as assimilation of nitrate (nitrate reductase, glutamine synthetase, aspartate aminotransferase, etc.) [123]. A similar observation was also reported by Ertani and co-workers [124], who found higher nitrate reductase and glutamine synthetase activity after receiving PHs treatment; leading to higher nitrate assimilation in roots and leaves of corn seedlings. Foliar application of SWEs were reported to enhance foliar nitrate reductase and *trans*-zeatin riboside (a cytokinin) levels in bentgrass [125]. A recent study reported that SWEs (extracts with some modifications) of *A. nodosum* upregulated the nitrate transporters’ (NRT1.1, NRT2.1, NRT1.5) gene expression and some other associated N assimilation enzymes in spring barley roots, thereby enhancing NUE; barley yield was maintained despite using 27% less N fertilizer under field conditions [62,126]. Interestingly, the stimulatory action of SWEs was observed to be more pronounced when plants were grown at lower nitrate levels, which implied that SWEs application might be a suitable strategy to enhance nutrient assimilation under nutrient-limited conditions commonly encountered in many organic farming scenarios [127]. Apart from the normal conditions, AMF and PGPR microbial BSs were reported to increase nitrate reductase activity in lettuce under moderate drought stress conditions [128]. Therefore, BSs appeared to be more effective for plants encountering sub-optimal conditions such as nutrient deficiency and adverse climatic conditions. 

## 4. Implications of Biostimulants for Enhancing Crop Physiology, Productivity, and Quality

Plant growth and yield are influenced by a variety of complex genetic, biochemical, metabolic, and environmental factors that are regulated by internal and external stimulators [7,9,21,29,74,80,86,129,130,131]. Due to the large diversity of BSs and complexity associated with plant growth and developmental regulation, it is likely that there will be several mechanisms regulating growth, development and responses to various stressors [28,34,42,43,74,80,86,129,131,132]. For a specific group of BSs such as vermicomposts and their various “teas,” the scientific evidence is indicative that vermi-linked BSs enhanced plant growth and development through bio-stimulatory actions of various phytohormones present in it [28,70,75,81,130]. One could envisage that various groups of BSs would employ different mechanisms in accordance with their intrinsic chemistry and molecular mechanisms.

In general, BSs promote growth, development, and resilience to abiotic stresses by exerting a bio-stimulatory effect on target plant due to phytohormones, secondary metabolites, and organic and inorganic nutrients [28,42,66,94,95,129,131]. Furthermore, application of BSs was reported to stimulate seed germination, seedling growth, and crop productivity by promoting primary and secondary metabolisms through the bio-stimulatory actions of signaling bioactive molecules existing in BSs [21,29,34,75]. A recent study by Briglia and coworkers [132] reported that BS application upregulated several genes involved in hormone metabolisms and biosynthesis, regulating nitrogen metabolisms and mineral transport in maize. Interestingly, researchers have reported that BSs effectively enhanced yield, nutritional and functional attributes of a wide range of fruits and vegetables [112,133,134]. *Trichoderma*-based BSs were reported to enhance biosynthesis and accumulation of phytochemicals such as ascorbic acid by regulating secondary plant metabolism, providing several health benefits to consumer [38,94,95,112]. Similarly, Carillo and co-workers [134] demonstrated that application of BSs on plum tomatoes enhanced the levels of lycopene, asparagine, and γ-aminobutyric acid by stimulating secondary metabolism and enhancing nutritional quality of fruits. Furthermore, AMF was able to modulate plant secondary metabolites’ synthesis, thereby improving the health-promoting attributes of fruits and vegetables [135]. A recent study by Di Mola and co-workers [136] indicated that legume-derived PHs were able to enhance antioxidant contents of green leafy vegetables, thereby improving the vital health benefits in consumer diet. Their study further revealed that PHs could further modify primary and secondary metabolism in spinach, thus contributing to various phytochemicals associated with numerous health-promoting attributes. Foliar application of SWEs (*A. nodosum*) was reported to increase health-promoting phenolics and flavonoids [137]. Moreover, Sani and co-workers [138] stated that both microbial and non-microbial BSs assisted in modifying primary and secondary metabolisms that led to synthesis and accumulation of antioxidants associated with health benefits. The combined application of *Trichoderma* + SWEs (*A. nodosum*) enhanced growth, nutritional quality, and mineral contents of organically grown tomatoes [138]. Similarly, a combination of *Trichoderma*-based BSs and bio-fortified spent mushroom substrate (SMS) improved nutritional quality of tomato through the synthesis and higher accumulation of TSS (total soluble sugars), carotenoids, polyphenols, and mineral contents [139]. Moreover, a plethora of recent research documented numerous beneficial effects of BSs on growth, physiology, yield, and quality, summarized in Table 1.

## 5. Implications of Biostimulants in Alleviating Stress in Crop Plants

Unfavorable climatic and soil conditions such as drought, salinity, and extreme temperature cause significant yield reduction in crops and are responsible for nearly 70% yield differences as determined by global climate analysis [174]. The global climate is predicted to change considerably in the next few decades and is likely to intensify adverse climatic extreme events affecting crop production and global food security [1,4,20,21,29,32,95]. In that context, non-microbial and microbial BSs have been widely touted as a promising technology to improve crop productivity and maintaining yield stability under adverse climatic conditions [21,29,32,100]. Although many BSs were able to enhance nutrient uptake, recent literature also reported that BSs could stimulate other rhizospheric microbes and plant-microbe beneficial associations, altering various metabolic and physiological mechanisms that allow them to ameliorate stress-induced adverse effects (Table 2). The stress adaptation strategies delivered by applications of microbial BSs included cell wall alteration and accumulation of high soluble solutes, leading to enhanced water retention, thereby improving the osmotic and ionic stress tolerance [95,98,113,129,175]. Interestingly, *Rhizobium* was shown to alleviate salt stress through the production and bio-stimulatory actions of high indole-3-acetic acid (IAA) and EPS concentrations [176]. These mechanisms were also reported to alleviate extreme temperature and drought stress in numerous agricultural and horticultural crops. Furthermore, inoculation of *Azotobacter* strains increased K+ uptake and exclusion of Na+, which mitigated the negative impact of salinity stress in wheat, ensuring increased biomass and grain yield [177]. For rain-fed field crops, inoculation of *Pseudomonas putida* resulted in significant improvement in heat tolerance of wheat by minimizing ROS generation [178]. Similarly, the cold-tolerant PGPR *Pantoea dispersa* was able to improve cold tolerance in wheat by enhancing nutrient solubilization and higher production of IAA [179]. *Burkholderia phytofrman,* another commonly used PGPR, was reported to increase ROS scavenging metabolites and stress-induced genes, thereby enhancing the chilling tolerance capacity in *Vitis vinifera* L. [180]. Interestingly, PGPR with ACC-deaminases were able to minimize ethylene-induced root inhibition, maintaining a higher root-to-shoot ratio and, consequently, achieving better growth under salt stress [181].

A recent study reported that non-microbial BSs such as SWEs were able to confer chilling stress tolerance in maize by enhancing the ROS responses through supplying micronutrients (Zn, Mn, etc.) [182]. Similarly, SWE-based cytokinins were reported to enhance heat tolerance in bentgrass (*Agrostis stolonifera* L.) [183]. The PHs derived from alfalfa showed better salt tolerance in maize through bio-stimulatory actions of triacontanol (TRIA) and IAA, which resulted in a higher concentration of flavonoids, proline, and potassium [184]. PHs containing higher amino acids showed antioxidant and free radical scavenging properties in lettuce and improved root dry biomass and yield and higher levels of osmolytes and glucosinolates [35]. The application of HSs in common beans showed increased endogenous proline concentration and minimal membrane leakage, facilitating better salt tolerance in plants [49]. Apart from beneficial actions/effects of BSs on abiotic stress tolerance, several studies also reported the role of BSs in biotic stress tolerance, especially for microbial BSs, although protection against biotic stresses generally does not fall under the arbitrarily accepted definition of BSs [30,102,103,128]. However, their potential bio-stimulatory role in biotic stress tolerance, in addition to growth promotion, will be relevant for future development of novel BSs products. Some beneficial microbes are known to regulate induced systemic resistance (ISR) in plants by stimulating the immune system against a broad spectrum of pests and providing more rapid and intense actions against pathogens without compromising growth and yield [185]. Among the microbial BSs, PGPR *Pseudomonas*, *Serratia*, and *Bacillus*, and beneficial fungi *Trichoderma* spp. and *Piriformospora indica* have been well documented to induce ISR in various crops [186,187]. *Trichoderma harzianum* based commercial formulations (Trianum-Pfi) were reported to induce ISR and provide defense against soil pathogens [188]. Furthermore, ACC deaminase-producing PGPR were able to protect plants against bacteria, fungi, and nematodes by hindering symptomatic development and minimizing disease severity [189]. For instance, the ACC deaminase producing *Pseudomonas putida* UW4 was able to provide protection against *Pythium ultimum* in cucumber [190].

## 6. Exploiting Synergistic Biostimulatory Interactions among Biostimulants

As discussed earlier, the pursuit of organic farming is to reduce dependence on agrochemicals, particularly inorganic fertilizers (e.g., phosphorus), by improving nutrient availability and NUE while maintaining soil health, soil quality, and productivity [3,8,9,13,16,17,24]. Microbial and non-microbial BSs have been widely reported to improve growth and protect plants from both biotic and abiotic stresses. When selected correctly, an application of these BSs would exert desired effect(s) on plants that are facing multiple abiotic constraints such as nutrient limitations, drought, salinity, heat, and concomitant biotic stresses simultaneously in a typical organic production system. Therefore, the purposeful combinations of microbial and non-microbial BSs represent a promising strategy that synergistically provide multiple beneficial effects to optimize growth and stress tolerance while enhancing yield and quality in these “low input” scenarios [9,13,14,26]. However, a combination of microbial and non-microbial BSs may result in three plausible outcomes: additive, antagonistic, and synergistic effects based on their interactive bio-stimulatory actions and mechanisms. Firstly, for additive effects, the combined effects exerted by BSs equaled the sum of their individual effects. Secondly, for antagonistic effects, the overall effect exerted by BSs delivered less than the additive effects. Lastly, synergistic effects could be observed when cumulative effects of BSs exceeded their additive effects; which is ultimately the preferred outcome. In recent years, many studies demonstrated that combined application of microbial and non-microbial BSs generally provided better benefits due to synergistic interactions among the BSs, resulting in enhanced growth, and stress protection [24,26,222,223,224,225]. For instance, microbial BSs *Trichoderma virens* (TG41) with a vegetal biopolymer-based BSs (VBP) enhanced CO_2_ assimilation in lettuce and increased mineral contents by 10% for K and 12% for Mg [226]. Their study further reported that a combined application of (TG41) + (VBP) interacted synergistically and enhanced the nutritional quality of lettuce by significantly increasing antioxidant activity, total ascorbic acid (+61–91%), and total phenols (+14%) while minimizing nitrate content. According to Sani and co-workers [138], a combined application of *Trichoderma*-based BSs and SWEs interacted synergistically and enhanced the growth, nutritional, functional quality (ascorbic acid, lycopene, minerals) of organically grown tomato. In addition, they found favorable synergistic interaction between *Trichoderma* and SWEs and the concomitant increase of soil fertility by fostering growth of rhizospheric fungal and bacterial populations, thereby increasing NUE, plant growth and with higher levels of antioxidants and minerals in their tomato experiments.

A recent study also demonstrated that AMF + SWEs induced a favorable synergistic effect; higher biomass, leaf area, stomatal conductance, mineral concentration (N and P) were reported in date palm [227]. Apart from optimal conditions, few studies also reported the synergistic interactions providing better resilience in stress conditions. A combination of plant-derived PHs and microbial BSs interacted synergistically and delivered a marketable yield of greenhouse lettuce (*Lactuca sativa* L.) under alkalinity and salinity stress [26]. The combination of AMF + SWEs resulted in producing an additive effect in increasing root growth as well as protein and carbohydrate content of tomato [166]. Interestingly, their study also found a synergistic effect in accelerating flowering of tomato plants and further demonstrated that additive and synergistic effects were due to the interactions between microbial (AMF) and non-microbial (SWEs) BSs, thereby delivering better plant performance. Researchers working on acclimatizing pineapple plants in a greenhouse found that humic acid and PGPR (*Burkholderia* spp.) increased 50% and 81%, respectively, whereas their combined application resulted in achieving the best growth (105%) [218]. The application of non-microbial BSs with AMF significantly increased the phenolic compounds, lipids, sugars, and proteins in leaves of *Moringa oleifera,* thus enhancing their functional properties [228]. A greenhouse study on wall rocket (*Diplotaxis tenuifolia* L.) demonstrated that combined application of PHs+ *Trichoderma harzianum* T22 interacted synergistically and increased lipophilic and hydrophilic antioxidant activity as well as ascorbic acid and chlorophyll content [229]. The study also found that combined application (PHs+ *Trichoderma harzianum* T22) also increased N, P, Mg, and Na contents compared to the stand-alone applications. Similarly, a combination of *Trichoderma harzianum*+ Biopolymer-based BSs enhanced crop performance, nutritional and functional quality of greenhouse-grown tomato [134]. A greenhouse study on perennial wall rocket demonstrated that PHs and BSs (tropical plant extracts) interacted synergistically and significantly promoted ascorbic acid content over the stand-alone applications [230]. In addition, it was reported that combined application of SWEs and PGPR interactions led to a significant increase in growth and photosynthetic pigments in *Amaranthus hybridus* [231].

The application of combined AMF and potassium humate BSs on Russian olive (*Elaeagnus angustifolia* L.) exhibited synergistic interactions and enhanced the antioxidant defense system through increasing superoxide dismutase and glutathione reductase activity as well as phenolic content [232]. A single application of HSs and SWEs increased groundnut plant height by 34.5% and 17.2%, respectively, whereas their combined application resulted in 65% compared to the sum of independent, stand-alone applications [225]. Alginic acid, a major component of SWEs, was able to promote hyphal growth in AMF, leading to enhanced P availability and improved nutrient uptake in plants [65]. Furthermore, HSs and AMF showed significantly increased root dry weight in onions by 43.9, and 12.1%, whereas their synergistic effect exhibited 106.7% compared to a sole application under elevated CO_2_ [223]. Their study suggested that the synergistic bio-stimulatory interactions of HSs and AMF resulted in achieving higher NUE, thus enhancing onion plants’ performance under elevated CO_2_. Similarly, a co-application of substrate with AMF (*R. intraradices*) and providing a subsequent HA spray at 30-day intervals delivered enhanced root biomass as well as greater chlorophyll biosynthesis compared to stand-alone applications in perennial ryegrass [224]. Moreover, Rouphael and co-workers demonstrated that endophytic fungal consortium and PHs improved crop productivity over a single application by increasing chlorophyll biosynthesis and maintaining the photosynthetic activity of PSII and leaf nutritional status [26]. In a field grown-tomato trial, a consortium of fungal and bacterial BSs delivered positive and synergistic effects on uptake of certain essential mineral nutrients (K, Na, and Mn) from soil [233]. Based on the selection of literature provided, the synergistic properties among BSs are interesting and indicative of their complex biostimulation mechanisms in determining plant growth, performance and resilience. Thus, to develop the next generation of BSs with specific synergistic effects for enhanced crop growth, yield, quality, and resilience, we need to characterize the BSs individually and when they are used in a mixture. 

## 7. Ecological Considerations for Harnessing the Beneficial Functions of Biostimulants: Moving from Lab towards Successful Field Application

Plant responses would be affected significantly by global climate change in terms of above and belowground interactions with the growing environment and diversity of organisms in terrestrial ecosystems. Therefore, applied BSs would need to be operative effectively in field conditions [4,7,21,24,29,51,100]. Several studies reported that efforts to use microbial BSs under field conditions have failed to improve crop performance consistently. The multiple-faceted interactions between plants and their symbiotic microbial species, ecological effects of plant-associated soil microbes and soil, and plant metabolic dynamics remain unclear [21,29,86,100,175,234,235]. For instance, we have little knowledge about the stand-alone microbial inoculants in soil after inoculation and how these inoculants interact with existing indigenous microbes while adapting to local abiotic conditions. Even with successful laboratory or greenhouse (pot, planting beds) trials, it is also unclear whether these introduced microbes could establish a compatible synergistic interaction with host plants, including aspects of molecular defense with the plant immune system under field conditions. Several factors can alter the success of microbial inoculation in agro-ecosystems, including plant-microbial compatibility, the degree of their competition with existing native microbial population, and timing of inoculation [29,100,175,236]. Throughout the whole growing period, this microbial community undergoes continuous interactions and succession with above- and belowground components of the crops [237]. Therefore, even if beneficial microbial inoculants colonize the plants and the adjacent soils initially, their persistence and functionality over time in the rhizosphere are not guaranteed. Moreover, measuring the persistence of these microbial inoculants in soil poses major technical limitations, as the inoculants need to be identified and profiled from within a complex community. In addition, a stress episode may also induce existing (local) microbes to produce a variety of compounds that may ultimately affecting the entire microbial community stability. For instance, it was reported that drought-treated soils contained more antibiotics, which were produced by drought-tolerant bacteria as a physiological response to outcompete other bacteria for limited resources or possibly acting as signals to induce drought-response pathways such as biofilm formation [238]. Therefore, the uncertainly posed by complex microbial and plant interactions on soil-microbiome functionality remained a challenge to the wider usage of BSs, especially under field conditions. Interestingly, attempts have been made along these similar lines in restoration ecology where researchers used various combination of BSs and an N-fixing legume (pigeon pea) to restore highly degraded mine site soils for the purpose of re-introducing native vegetation post-mining [239]. 

In this context, microbial and non-microbial BSs’ combined application may offer plants with better combinatorial effects through synergistic interactions favoring beneficial physiological functions to plants. For example, HSs were effective in enhancing germ tube elongation and hyphal branching of AMF, thus assisting the symbiotic expansion of AMF in onions and thereby boosting root and shoot biomass production [223]. Several studies demonstrated that combined application of AMF, *Trichoderma*, or PHs stimulated the uptake of bivalent cations, principally Mg^2+^ and Fe^2+^ that were required for chlorophyll biosynthesis and restoring foliar chlorophyll content to acceptable levels under adverse saline and alkaline field conditions [113,154]. Furthermore, Rouphael and co-workers [26] reported that a combined application of microbial-based BSs and PHs interacted synergistically to activate both proline and antioxidant enzymes as a strategy against oxidative damage under stress conditions and was proven more effective than a single microbial BS application. Several researchers [54,238] reported that PHs could possibly stimulate plant-associated microbiome; thus, these amino acids were serving as suitable substrates for plant-associated microbes in rhizosphere. Therefore, the determinants of plant productivity and stress responses under field conditions are dependent in part on diverse microbial communities in rhizosphere. Their underlying interactions with plants and understanding the combinatorial effects of BSs on soil microbiome function would pave the way to improve our understanding of the soil-microbe-plant continuum. With more targeted research in BS characterization and metabolism dynamics along the soil-microbe-plant continuum (e.g., BSs involved in root to shoot signaling after inoculation), we will better understand the communication dynamics of plants and microbes in rhizosphere [21,29,235,240]. With greater mechanistic clarity, we may be able to access the beneficial potential of these plant-microbe interactions through strategic BS usage. 

## 8. Concluding Remarks and Future Challenges

Microbial and non-microbial BSs offer a promising innovative and sustainable strategy to supplement and replace agrochemicals in the near future. With greater mechanistic clarity, the judicious use of BSs should improve plant growth and resilience to biotic and abiotic stresses and deliver acceptable yield and good quality organically cultivated products. The first step is to understand and characterize the diversity of BSs using advanced analytical approaches with concomitant validation of plant performance over a wide range of conditions. The research community, growers and industrial companies are interested in identifying the bioactive elements of BSs and elucidating underlying biochemical, physiological, and molecular pathways of biostimulation. If the characterization of targeted BSs is successful, we will be able to formulate more specific BSs to meet specific species requirements and address the multitude of cultivation challenges. Nevertheless, further advanced research is needed to address several uncertainties, such as: (i) How effectively can BSs modulate the rhizospheric microbial population quantitatively and qualitatively in rhizosphere? (ii) How long can microbial BSs persist under field condition subsequent to their application and their underlying interactions with existing microbes? (iii) How do BSs modulate hormonal signaling under both normal and stress conditions within a plant? (iv) How and to what extent BSs stimulate microbe-derived hormones in root microbiome assembly, rhizosphere, entry to plant vascular system, and root-shoot signaling? The recent advancements in omics-based and other technologies, such as meta-transcriptomics, meta-proteomics or metabolomics, amplicon sequencing and phenotyping, will contribute to profiling of trace metabolites facilitating the soil-microbe-root-shoot processes, and consequently help assess plant performance and yield. Understanding these mechanisms will lead to the development of novel and well characterized BSs, combining microbial and non-microbial BSs strategically with specific desired synergistic bio-stimulatory action, to deliver enhanced plant growth, yield, quality, and resilience consistently in organic agriculture.

## Figures and Tables

**Table 1:** Biostimulatory effect of biostimulants in enhancing crop physiology, productivity, and quality.

BSs Applied	Crop	Effect on Crop Growth, Yield and Quality	Reference
SWEs (*Ascophyllum nodosum*)	Wheat	Increased in grain yield and protein quantity	[140]
SWEs (*E. maxima*, *A. nodosum*, *Sargassum* sp.)	Tomato	Increased mineral (Fe, Zn) content, enhanced germination, plant height, chlorophyll content, yield Expression of 6 flowering genes, increased flower bud and fruits	[141,142,143]
SWEs (*Sargassum swartzii*)	Cowpea	Increased phenolic and flavonoid content	[144]
SWEs (*A. nodosum*, *Laminaria ochroleuca*)	Broccoli	Increased antioxidants, flavonoids, and phenolic Enhanced both glucosinolates and phenolic compounds	[145,146]
SWEs (*E. intestinelis*)	Cucumber	Increased mineral (Fe, Mn, Zn) content of fruits, yield	[147]
SWEs (*A. nodosum*)	Pepper	Increased growth (height), chlorophyll content, yield	[148]
SWEs (*Ecklonia maxima*)	Spinach	Increased leaf number, chlorophyll, carotenoids, proteins, phytohormones, and phenolic acid	[149]
SWEs (commercial mixture)	Maize	Enhanced carbohydrate, organic substance and phosphorus metabolism, increased PGPR in rhizosphere	[150]
SWEs (*A. nodosum*)	Strawberry	Increased 10% marketable yield	[151]
SWEs (*Ecklonia maxima*)	Common bean	Increased yield and anthocyanins content in the seeds Increased synthesis of phenolics, flavonoid, anthocyanins and antioxidant activities	[152,153]
HSs	Maize	Increased leaf biomass, chlorophyll and carotene content Increased growth, grain yield and water use efficiency Faster induction of a higher capacity to take up nitrate	[154,155]
HSs	Onion	Increased yield, carbohydrate, protein and mineral contents in bulb	[156,157]
HSs	Strawberry	Increased growth, nutritional and chemical composition	[158]
HSs	Common bean	Increased seed yield and mineral content	[159]
HSs	Thai basil	Increased leaf nitrogen content	[160]
HSs	*Arabidopsis*	Enzyme activation of the glycolytic pathway and up-regulation of ribosomal protein	[161]
PHs	Tomato	Increased photosynthesis, antioxidant activities, total soluble solids, mineral composition Regulated the expression of genes involved in nitrate, ammonium and amino acid transporters as well as the key genes involved in N metabolism	[162]
PHs	Maize	Increased macro-and micro-nutrients in leaves, protein content in grain and yield Increased growth and accumulation of N-compounds (proteins, chlorophylls and phenols) Increased root growth and accumulation of K, Zn, Cu, and Mn in roots	[163,164,165]
AMF	Tomato	Increased foliar and root growth and protein content	[166]
AMF	Maize	Increased biomass and yield through biological improvement of soil properties	[167]
*Trichoderma*-based BSs	Lettuce, Rocket	Increased growth, yield and nutritional quality	[38,138,168]
PGPR (*Bacillus* spp.)	Tomato	Increased growth and yield	[169]
PGPR (*Bacillus amyloliquefaciens*)	*Arabidopsis*	Increased photosynthesis, biomass and seed yield	[170]
PGPR (consortia)	Wheat	Increased root growth and nitrogen accumulation	[171]
PGPR (*Cellulosimicrobium* and *Pseudomonas*)	Pepper	Increased phenolic compounds	[172]
PGPR (*Azospirillum* and *Agrobacterium*)	Pea	Increased nutrient uptake, vegetative growth, chlorophyll content and antioxidant capacity	[173]

Abbreviations: AMF, Arbuscular mycorrhizal fungi; BSs, Biostimulants; HSs, Humic substances; PGPR, Plant growth-promoting rhizobacteria; PHs, Protein hydrolysates; SWEs, Seaweed extracts.

**Table 2:** Biostimulatory effect of biostimulants in enhancing stress tolerance and crop performance.

BSs Applied	Type of Stress	Crop	Effect on Stress Tolerance and Crop Performance	Reference
SWEs (*Euglena gracilis*)	Drought/water stress	Tomato	Increased antioxidants (carotenoids, vitamins and phenolic acids) and soluble carbohydrates (glucose, fructose, and sucrose) in fruits;Increase endogenous indole-3-acetic acid (auxin), *trans*-zeatin (cytokinin), and jasmonic acid	[191,192]
SWEs (*A. nodosum)*	Drought	Soybean	Reduced Reactive Oxygen Species (ROS), increased antioxidant enzymes activity, stomatal conductance, higher energy efficiency	[193]
SWEs (Commercial)	Cold	*Arabidopsi*s	Increased superoxide dismutase activity in the root and leaf tissue	[194]
SWEs (*Gracilaria dura*)	Drought	Wheat	Increased abscisic acid content and expression of stress-protective genes	[195]
SWEs (*A. nodosum)*	Drought	Spinach	Increased leaf-water relations, growth and yield	[196]
SWEs (*A. nodosum)*	Drought	*Arabidopsis*	Enhanced stomatal conductance and water use efficiency; regulation of stress-responsive genes	[197,198]
SWEs (*A. nodosum)*	Heat	Tomato	Gene transcription of protective heat shock proteins and increased flowering and fruit number	[199]
SWEs (*A. nodosum)*	Drought	Broccoli	Increased N, P, K, Mg, Cu and Mn contents	[200]
HSs	Drought	Potato	Increased growth, photosynthetic capacity and fresh tuber yield	[201]
HSs	Heavy metal stress (Cd)	Wheat	Increased activation of superoxide dismutase (SOD), catalase (CAT) and NADPH-oxidase (NOX) enzymes and ascorbate, glutathione	[202]
HSs	Salt	Strawberry	Enhanced leaf water content, membrane stability, chlorophyll content and increased biomass and yield	[203]
HSs	Drought	Rapeseed	Improved plants net photosynthesis via increasing the rate of gas exchange and electron transport flux	[204]
PHs	Salt	Common bean	Increased leaf photosynthetic pigments contents, membrane stability, relative water content	[205]
PHs	Drought	Grapevine	Reduced water loss, enhanced yield and quality	[206]
PHs (legume derived)	Mineral nutritional Stress (N)	Baby lettuce	Increased fresh weight, antioxidant capacity and total ascorbic acid content	[207]
PHs (legume derived)	Mineral nutritional Stress (N)	Baby rocket	Increased lipophilic antioxidant activity and total ascorbic acid content	[208]
PHs (legume derived)	Mineral nutritional Stress (N)	Baby spinach	Increased lipophilic and hydrophilic antioxidant activities, higher leaf chlorophylls and lower nitrate content	[136]
*Trichoderma* based BSs	Mineral nutritional stress (N)	Rocket	Improved root N uptake; increased ascorbic acid, K and Ca contents	[38]
AMF	Drought	Fenugreek	Increased root fresh weight, fresh plant weight and seed yield	[209]
AMF	Salt	Wheat	Increased photosynthesis and stomatal conductance, lower intrinsic water use efficiency and grain yield	[210]
AMF	Salt	Sweet basil	Increased chlorophyll content, water use efficiency and yield	[211]
AMF	Drought	Maize	Increased photosynthesis, proline, sugars and free amino acids; up-regulation of the antioxidant defense system	[212]
AMF	Heavy metal stress	Soybean	Retained heavy metals in roots and reduced translocation of Cu, Pb and Zn and improved overall growth and seed yield	[213]
PGPR (*Pseudomonas fluorescens* and *Microccucuce yunnanensis*)	Mineral nutritional stress (Fe)	Quince	Enhanced the expression of the genes related to Fe homeostasis, increased root, shoot biomass and chlorophyll content	[214]
PGPR (*Cupriavidus necator* and *Pseudomonas fluorescens*)	Water stress	Maize	Increased N and P use efficiency and biomass	[215]
PGPR (*Pseudomonas aeruginosa* and *Burkholderia gladioli*)	Heavy metal stress (Cd)	Tomato	Alleviated Cd toxicity and enhanced phenolic compounds, organic acids and osmoprotectants	[216]
PGPR (*Enterobacter* HS9 and *Bacillus* G9)	Water Stress	Velvet bean	Improved total biomass, water use efficiency and carbon assimilation	[217]
PGPR (*Alcaligenes faecalis*)	Salt	Wheat	Improved ionic balance, increased accumulation of osmolyte, photosynthetic pigments and improved photosystem II efficiency	[218]
PGPR (*Azospirillum brasiliense* and *Azotobacter chroococcum)*	Salt	Coriander	Increased chlorophyll content, fresh weight and yield	[219]
PGPR (*Bacillus licheniformis* and *Pseudomonas plecoglossicida*)	Salt	Sunflower	Increased fresh and dry biomass, yield, enhanced up-regulation of catalase (CAT), superoxide dismutase (SOD) and guaiacol peroxidase (GPX) antioxidant enzymes	[220]
PGPR (*Streptomyces* spp.)	Drought	Tomato	Increased leaf RWC, proline, MDA, H_2_O_2_ and total sugar content and yield	[221]

Abbreviations: AMF, Arbuscular mycorrhizal fungi; BSs, Biostimulants; HSs, Humic substances; PGPR, Plant growth-promoting rhizobacteria; PHs, Protein hydrolysates; SWEs, Seaweed extracts.

## Data Availability

Not applicable.
